# Efficacy of Episil® in patients with hematologic malignancies: a comparative study

**DOI:** 10.1186/s12903-024-04233-6

**Published:** 2024-05-03

**Authors:** Taeko Fukutani, Yukio Yoshioka, Shinpei Imori, Hirokazu Yanagihara, Kensaku Sumi, Yoshinari Myoken, Yoshinori Fujita, Souichi Yanamoto

**Affiliations:** 1 Department of Oral Oncology, Graduate School of Biomedical and Health Sciences, Hiroshima University, 1-2-3 Kasumi, Minami Ward, Hiroshima, 734-8553 Japan; 2 Graduate School of Advanced Science and Engineering, Hiroshima University, 1-3-1 Kagamiyama, Higashi-Hiroshima, Hiroshima, 739-8526 Japan; 3 Department of Oral Surgery, Hiroshima Red Cross and Atomic-Bomb Survivors Hospital, 1-9-6 Sendamachi, Naka-Ku, Hiroshima, 730-8619 Japan

**Keywords:** Episil® oral liquid, Oral mucositis, Hematologic malignancy, Speech function, Pain relief

## Abstract

**Background:**

Episil® is a nonabsorbable liquid medical material used to coat and protect the mucosa in patients with oral mucositis. A few studies have reported its efficacy in patients with head and neck cancer. However, reports on its use in patients with hematologic malignancies are scarce. Thus, we aimed to evaluate the efficacy of Episil for the treatment of oral mucositis in patients with acute myelogenous leukemia, malignant lymphoma, acute lymphocytic leukemia, multiple myeloma, and myelodysplastic syndrome.

**Methods:**

Between May 2018 and March 2019, a total of thirty-seven patients with acute myelogenous leukemia, malignant lymphoma, acute lymphocytic leukemia, multiple myeloma, and myelodysplastic syndrome who received Episil® for the treatment of oral mucositis were included in this study. All patients were treated at the Hiroshima Red Cross and Atomic-bomb Surgery Hospital. To determine the severity of oral mucositis, 22 out of the 37 patients were interviewed and compared objectively using the Common Terminology Criteria for Adverse Events, version 3.0. In addition, subjective measures of the effects of oral mucositis were assessed using an original evaluation protocol (a unique evaluation chart specific to the Department of Oral Surgery, Hiroshima Red Cross & Atomic-bomb Survivors Hospital).

**Results:**

Out of 37 participants recruited in the study, 31 (84%) described the sensation of Episil® as very good or good. Moreover, the severity of mucositis was found to decrease after the use of Episil® in seven patients out of 22 (19%), particularly in those with mucositis at multiple sites. Participants' evaluations revealed pain relief and improvement in speech and feeding functions. Participants with grade 3 mucositis reported a greater improvement in pain relief, speech, and feeding functions than those with grade 2 mucositis.

**Conclusions:**

This study suggests the efficacy of Episil® in treating oral mucositis in patients with hematologic malignancies, particularly in those with oral mucositis at multiple sites. In addition to pain relief, Episil® may improve speech and feeding functions.

**Supplementary Information:**

The online version contains supplementary material available at 10.1186/s12903-024-04233-6.

## Background

Oral mucositis (OM) is a common adverse event observed in patients undergoing chemotherapy, radiation therapy involving the oral cavity, and hematopoietic stem cell transplantation (HSCT) for the treatment of malignancies. The oral cavity plays various roles directly related to life functions, such as eating and speaking. Poor oral and swallowing function can lead to increased systemic vulnerability and decreased physiologic reserve [1–3]. The pain associated with OM can significantly interfere with feeding, necessitating tube feedings and the intravenous administration of fluids, increased use of opioids, and discontinuation of malignancy treatment [4, 5]. Thus, refractory OM and OM at multiple sites can diminish the quality of life, prolong treatment of the underlying disease, and shorten the patient’s life span. The incidence of OM has been reported in at least 50% of patients with solid tumors undergoing chemotherapy [6]. Patients with OM frequently experience significant pain and difficulty in feeding, swallowing, and speaking. This leads to a reduction in food intake and significant physical and emotional distress. The decline in nutritional status and quality of life can also impede the treatment of the underlying disease, which is a major concern for healthcare providers.

The incidence of oral complications increases as the use of molecularly targeted agents increases. In addition, their management also becomes more challenging [7]. Previous reports on OM have focused on pain, feeding function, and nutritional status. Although several systematic reviews have focused on OM, literature regarding its effect on speech function remains scarce [8–10]. Speech, the most important oral function, is used for communicating with healthcare providers and family members and is thought to play a key role in motivating patients to seek treatment. Evidence regarding the use of many available approaches to prevent, mitigate, and treat OM is scarce [7]. Moreover, no true evidence-based clinical practice guidelines have been published for the treatment and prevention of OM [11].

Therefore, the Multinational Association of Supportive Care in Cancer and the International Society for Oral Oncology (MASCC/ISOO), two international societies for supportive care in cancer, developed and published the first guidelines for the prevention of mucosal damage associated with cancer treatment in 2004 using a systematic review approach [12]. The revised version of these guidelines was published by the Mucositis Study Group of MASCC/ISOO in 2019–2020 owing to a significant increase in the number of studies on mucosal damage since the publication of the first guidelines in 2014 [13]. However, these guidelines did not recommend fundamental treatment for OM and mainly focused on coping therapies. In addition, as these are international guidelines, they often refer to drugs that are not approved in other countries or off-label use [14]. Therefore, this study focused on Episil® oral liquid.

Li and Trovato [15] reported that OM is observed in 97% of patients undergoing chemoradiotherapy for head and neck cancer, which is higher than that in other cancers. In contrast, OM has been observed in 98% of patients undergoing HSCT [16, 17]. The incidence of severe OM and OM involving multiple sites is inevitable in patients with hematologic malignancies for which high-dose chemotherapy and HSCT are indicated. Unlike patients with head and neck cancer, most patients with hematologic malignancies have neutropenia, and the use of topical steroid ointments is contraindicated owing to the increased risk of severe oral candidiasis. OM with severe neutropenia is frequently observed in patients with hematologic malignant diseases, such as leukemia and malignant lymphoma, undergoing chemotherapy and radiation therapy prior to bone marrow transplantation [18]. Advances in supportive care for infection, bleeding, and anemia have resulted in the intensification of chemotherapy with increased doses and frequency of administration for patients with hematologic malignancies. Although there are limited reports on the use of Episil® in patients with head and neck cancers, few studies have reported its use in patients with other malignancies, such as hematologic malignancies.

This study aims to demonstrate the efficacy of Episil®, which lacks drug components, in treating OM in patients with hematologic malignancies. The primary focus of this study is on enhancing speech and feeding function along with providing pain relief. We conducted a comparative analysis of Episil® efficacy by evaluating the severity of OM before and after usage in 37 patients with hematologic malignancies admitted to our hospital.

## Methods

### Episil® oral liquid

Episil® oral liquid is a bioadhesive, barrier-forming, non-absorbable, liquid medical device formulated to cover and protect eroded and ulcerated regions of the mucous membranes in the mouth, alleviating pain caused by the external stimulation of areas with OM. The use of Episil® was recently approved in Japan.

Episil® received European Community certification as a Class 1 medical device in May 2009 and was approved for use by the U.S. Food and Drug Administration in September 2011. As of March 2020, the use of Episil.® has been approved in 38 countries, including European countries and the U.S. [19].

Since 2018, Episil® has been available under insurance coverage in Japan for perioperative management of oral function. The primary component of Episil® includes the food additives soy phosphatidylcholine (SPC) and glycerol dioleate (GDO). Thus, it is classified as a medical device—not a drug—as it contains no drug ingredients. SPC and GDO react with moisture in the saliva, leading to structural changes into an adhesive gel. This gel adheres to the mucosal surface as a film after the lipid components self-assemble. The gel is believed to coat and protect ulcerated mucosa regions, thereby providing relief for pain. Given its non-drug formulation, Episil® is free from adverse effects. In addition, there is no risk of interaction with other drugs, making it advantageous for easy use as a supportive therapy.

### Study design

**Fig. 1:**
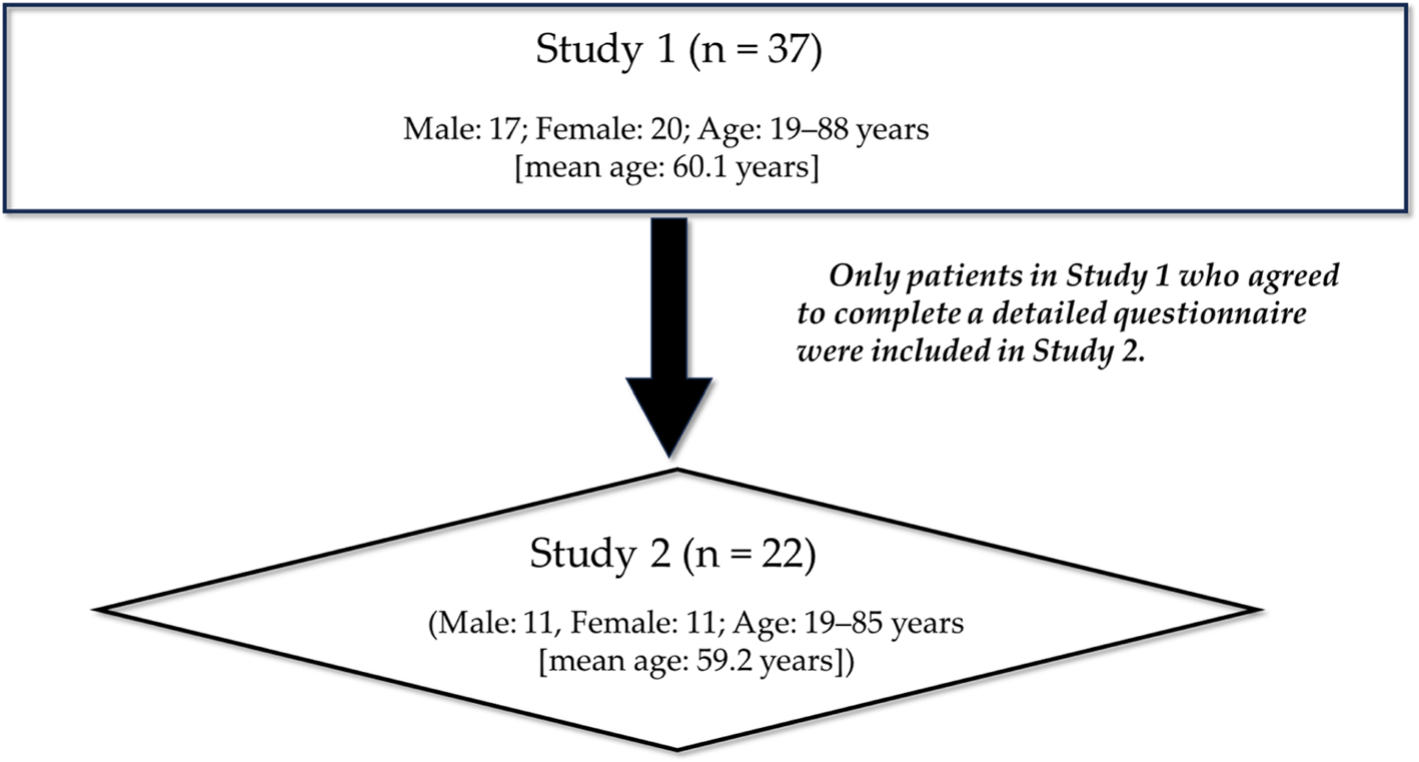
Illustrations depicting Study 1 and Study 2

This study was divided into two parts: Study 1 (*n* = 37) and Study 2 (*n* = 22) (Fig. 1). The participants were categorized into two groups. Group A comprised 37 participants, and Group B constituted a subset of 22 participants from Group A who completed the questionnaire.

#### Study 1

Study 1 included 37 participants (17 males and 20 females; age: 19–88 years [mean age: 60.1 years]) with hematologic malignancies and OM who sought treatment at the Department of Oral Surgery, Hiroshima Red Cross & Atomic-bomb Survivors Hospital between May 2018 and March 2019. Among these participants, 18 had acute myelogenous leukemia, 9 had malignant lymphoma, 7 had acute lymphocytic leukemia, 2 had multiple myeloma, and 1 had myelodysplastic syndrome. Neutrophil counts were normal (> 1500 cells) in 3 cases, neutropenic/mildly reduced (1000–1500 cells) in 1 case, moderately reduced (500–1000 cells) in 2 cases, and severely reduced (< 500 cells) in 31 cases (Table S1). The endpoints for this study were the experience with Episil® and number and sites of onset of OM.

#### Study 2

Study 2 comprised a subset of 22 participants from Study 1 who completed a detailed questionnaire (11 males and 11 females; age: 19–85 years [mean age: 59.2 years]). Among these participants, eight had acute myelogenous leukemia, six had malignant lymphoma, five had acute lymphocytic leukemia, two had multiple myeloma, and one had myelodysplastic syndrome. Neutrophil counts were normal in 2 cases, neutropenic/mildly reduced in 1 case, moderately reduced in 1 case, and severely reduced in 18 cases (Table S2). The endpoints for this study were the changes in OM level before and after Episil® application and oral function assessment and their degree of improvement.

### Study procedure

The package insert recommends applying a few drops of the solution to the affected area, spreading it with the tongue, and leaving it on for a few minutes until the formation of a film. However, challenges arose with this approach, including difficulties in removing the nozzle, uneven spreading of the liquid, and quick peeling of the internal liquid. Consequently, the investigation focused on refining the usage method, the tools for application, and the duration of the film retention after application. The goal was to standardize the formulation's use as much as possible.

Accordingly, the investigation was conducted after ensuring that the patient, nurses, and family members were acquainted with the following protocols:

1. The preparation should be applied with the aid of a nurse or family member.
2. The attending physician and their assistant should confirm the size and position of the affected area before the patient uses this preparation for the first time.
3. Excess water after brushing the teeth or rinsing the mouth should be removed using a gauze before application.
4. The formulation should be applied using clean or gloved fingers.
5. Mouth rinsing, eating, and drinking should be avoided for approximately 10 min after application. Episil® is spread over the oral mucosa via the pump bottle. According to the package insert of Episil®, one pressurization yields 0.15 ml of liquid, and it is recommended that 1–3 drops be used per dose thrice daily.
6. The product should be applied before and after meals and reapplied as necessary in cases of peeling or pain.

#### Study 1–1: Feedback from patients who used Episil®

Approximately seven days after using Episil®, a questionnaire survey was conducted to assess the user’s experience. The survey responses included: A) very good, B) good, C) no effect, and D) bad (indicating adverse events).

#### Study 1–2: Evaluation of OM severity before and after the use of Episil® and its comparison

The severity of OM was assessed and compared before and after the use of Episil® based on the criteria outlined in the Common Terminology Criteria for Adverse Events (CTCAE) Version 3.0 (Table 1).

**Table 1:**
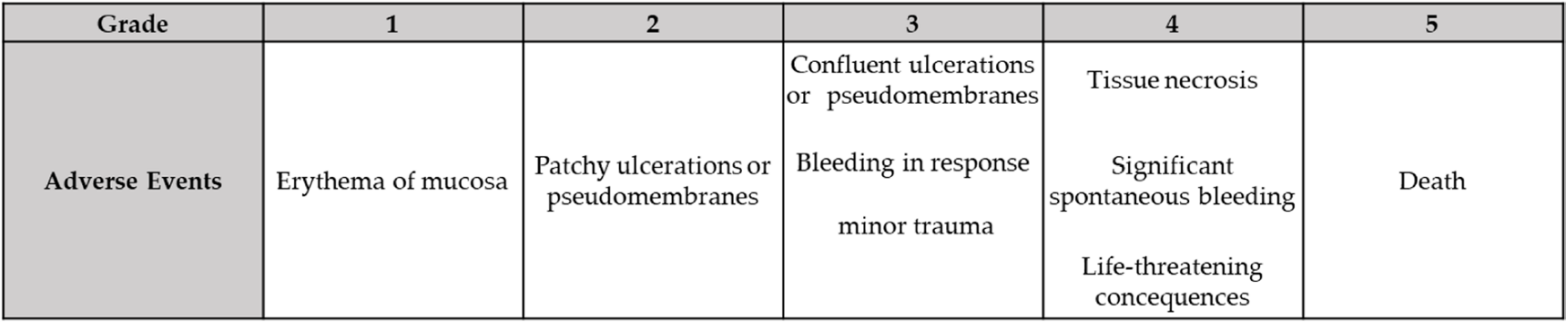
Adverse event criteria for OM in CTCAE Version 3.0

*CTCAE* Common Terminology Criteria for Adverse Events

#### Study 1–3: Examination of the relationship between the number of sites showing the onset of OM and OM grade in patients

Based on the findings of Study1-2, the association between the number of sites showing the onset of OM and OM grade was investigated using a one-tailed t-test for means with a paired sample.

#### Study 2–1: Comparative study of each oral function before and after the use of Episil®

A unique evaluation chart (Table 2) specific to the Department of Oral Surgery, Hiroshima Red Cross & Atomic-bomb Survivors Hospital, was developed to assess the effectiveness of Episil®. The severity of OM before and after Episil® use was compared to evaluate the efficacy of the formulation’s efficacy.

**Table 2:**
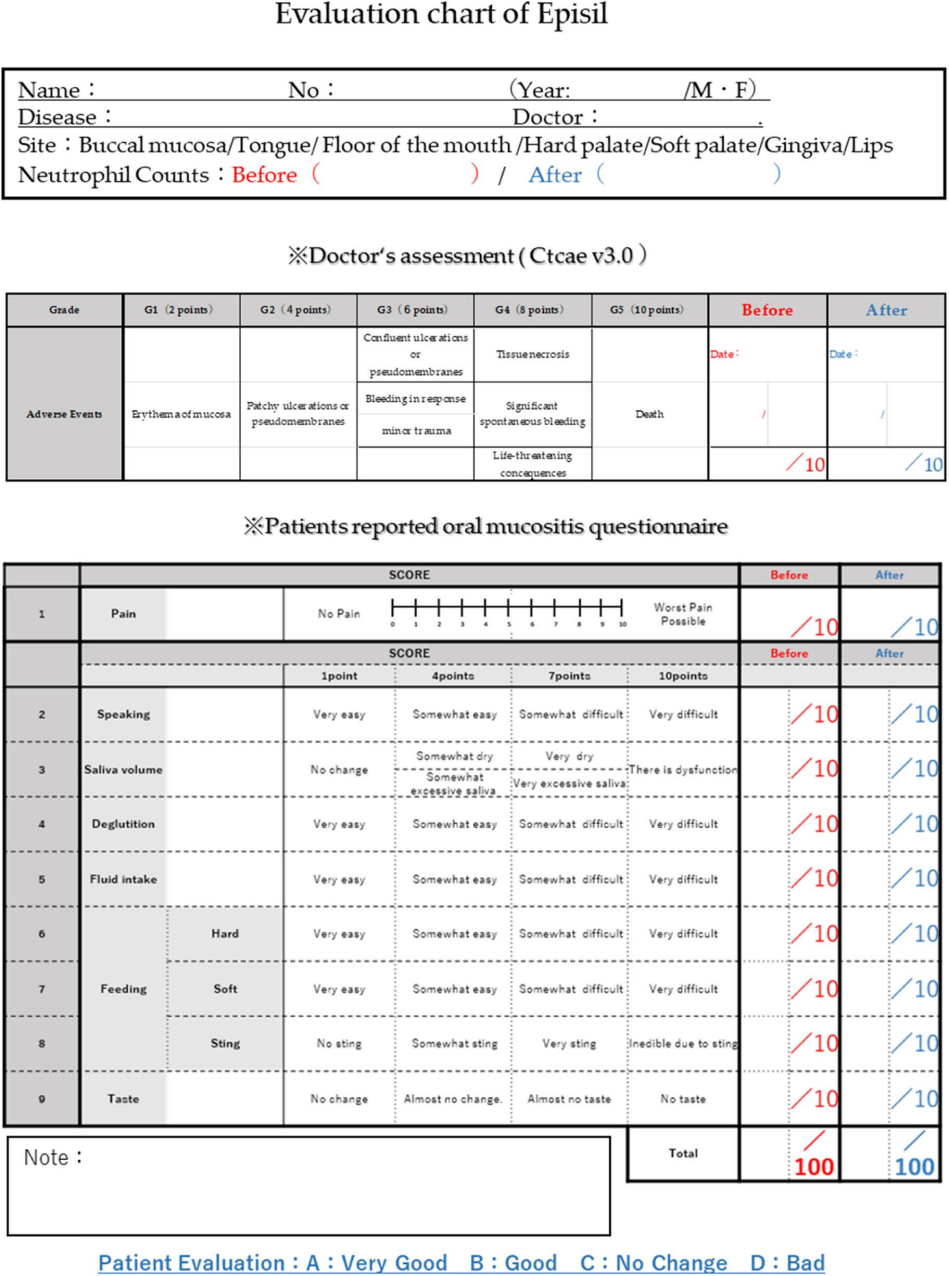
Department of oral surgery, Hiroshima red cross & Atomic-bomb survivors hospital original effectiveness evaluation chart

The evaluation chart consisted of two sections:

- Section A: Objective evaluation of OM by the attending physician on a 10-point scale based on CTCAE, Version 3.0.
- Section B: Subjective evaluation (90 points) by the patients.

Participants were instructed to rate (1) pain (assessed using the numerical rating scale), (2) speaking, (3) saliva volume, (4) swallowing, (5) fluid intake, (6) eating (hard food), (7) eating (soft food), (8) eating (sting), and (9) taste after the use of Episil®. Participants were asked to assign scores of 1, 4, 7, or 10. with the total score being the sum of Sections A + B = 100 points.

Section B was evaluated first. The scores for each item before and after the use of this formulation were evaluated using a t-test (one-tailed, test of means with a paired sample) to determine which of the functions in section B were the basis for the higher rating for Episil®. The overall score, including the score of A (A + B = 100 points), was subsequently analyzed using the same method.

#### Study 2–2: Comparison of the degree of improvement in OM and each oral function before and after the use of Episil®

Subsequently, Sections A and B of the evaluation chart were examined. The degree of improvement in the assessed values before and after using the formulation was evaluated based on the results of Study 2–1. Each item was transformed into a binary variable indicating whether improvement occurred before or after medication. The probability of improvement was assessed using statistical hypothesis testing to determine whether it was due to a chance. The probability of improvement after using the medication for each item was denoted as ‘p’, and the number of improvements after using the medication was modeled as a binomial distribution B(22, p) with H0: *p* = 0.5, H1: *p* > 0.5. A one-tailed test was conducted using the R statistical software [20] with the function ‘binom.test.'

### Ethical review

This study was conducted in accordance with the Declaration of Helsinki and approved by the Ethics Committee of the Hiroshima Red Cross & Atomic-bomb Survivors Hospital (approval no. 2019–080-2). Verbal informed consent was obtained from all participants.

### Statistical analyses

Study 1 and Study 2–1 used a t-test, while Study 2–2 was assessed using a statistical hypothesis test.

## Results

### Study 1–1

**Fig. 2:**
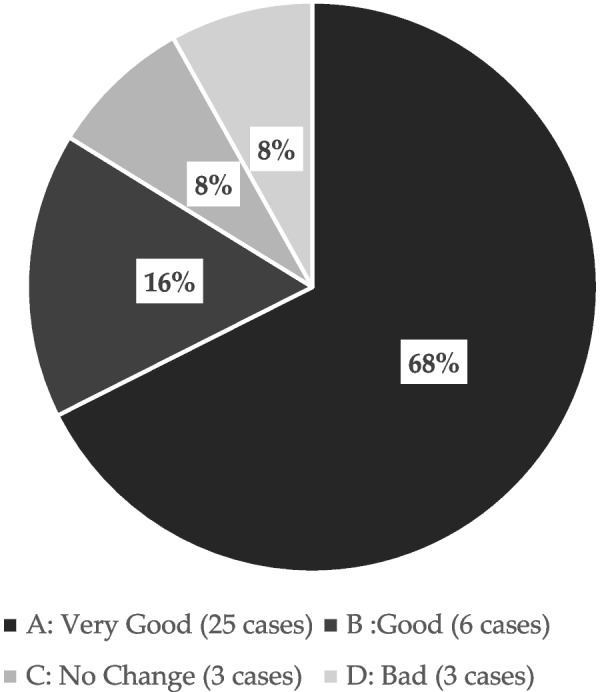
Findings from a survey on patient satisfaction with the use of Episil. In total, 84% of patients reported that their experience with Episil® was very good or good

The sensation of Episil® was described as A (very good), B (good), C (no effect), and D (bad) by 25, 6, 3, and 3 patients, respectively, with 84% of patients describing the sensation as very good or good (Fig. 2). Adverse events were reported in three patients. Sticking to the mucosa and pain were reported in two cases, and stinging was reported in one case.

### Study 1–2

**Fig. 3:**
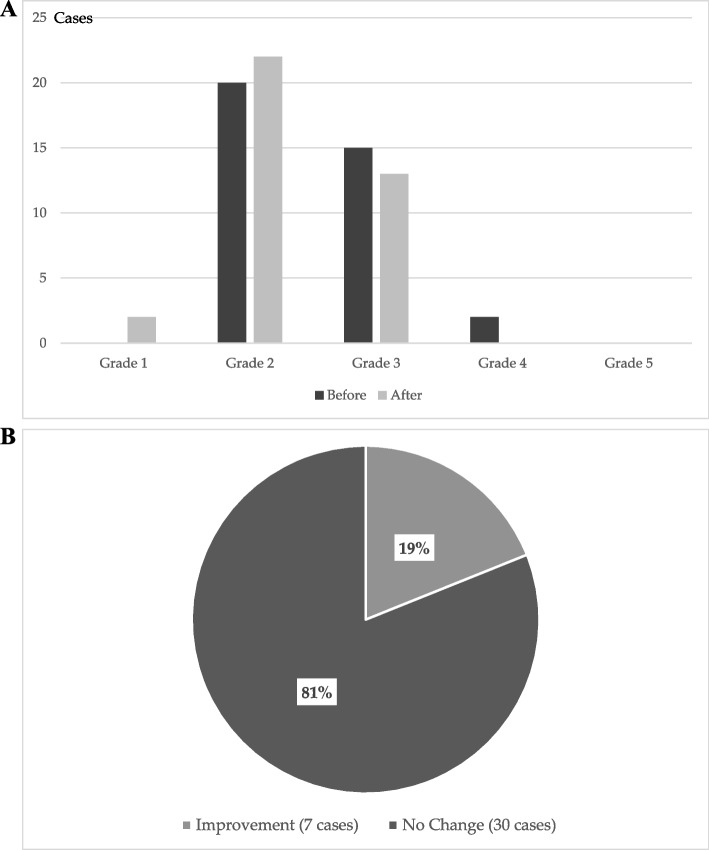
**A** Distribution of oral mucositis grades. **B** Changes in the severity of oral mucositis (OM) after the use of Episil®. The OM grade decreased in 19% of patients

Mucositis was evaluated using CTCAE, Version 3.0. The grade of OM was observed to decrease in 19% of patients after the use of Episil®. However, no change was observed in 81% of patients. Exacerbation of OM was not observed in any patients (Fig. 3A and B).

### Study 1–3

The site most frequently affected by OM was the tongue (26 patients), followed by the buccal mucosa (14 patients). Additional affected sites included the lips (12 patients), floor of the mouth (11 patients), soft palate (11 patients), gingiva (5 patients), and hard palate (5 patients) (Fig. S1).

**Fig. 4:**
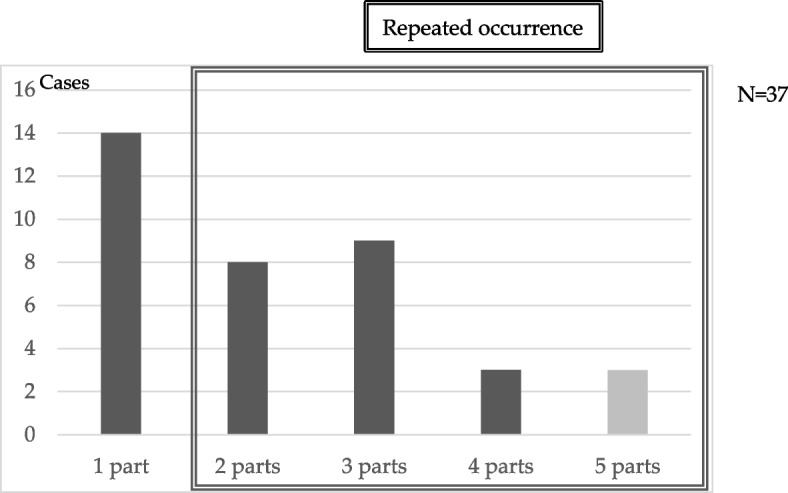
The number of oral mucositis (OM) lesion sites per patient. The occurrence of OM in five sites was associated with a decrease in OM grade

**Fig. 5:**
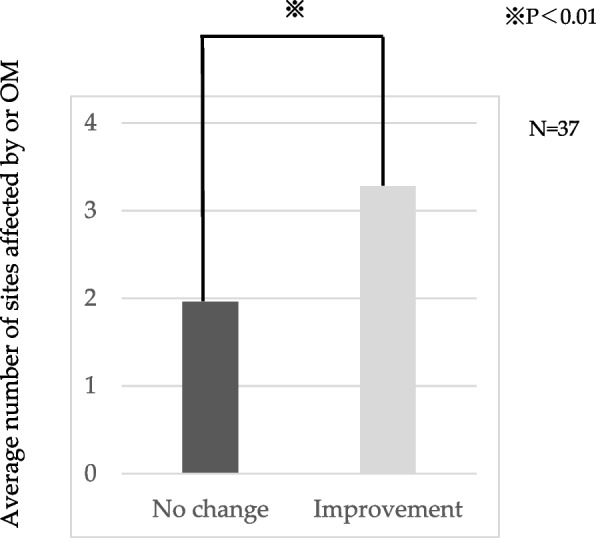
The correlation between the number of sites affected by oral mucositis (OM) and OM grade. There was a significant difference in the mean number of sites affected by OM between groups with unchanged and improved grades

OM was observed at multiple sites in 23 (62.2%) of the 37 patients (Fig. 4). Among these, three patients (patient nos. 21, 29, and 33) with OM at five sites showed a decrease in grades and improvements in OM. The *p*-value was 0.00493, indicating a significant difference in the mean number of sites affected by OM between the groups with unchanged and improved grades (Fig. 5).

### Study 2–1

**Fig. 6:**
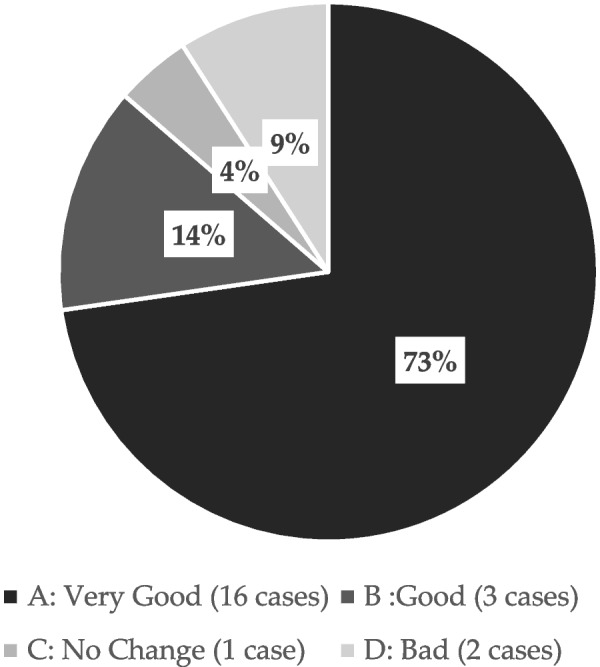
Results of a survey on the use of Episil®. In total, 87% of patients reported that their experience with Episil was very good or good

Among the 23 patients included in this study, 87% provided a response of very good or good, with 16, 3, 1, and 2 patients providing the responses A (very good), B (good), C (no effect), and D (bad), respectively (Fig. 6). The formulation was evaluated using the departmental evaluation chart. The patients’ subjective evaluations of B were examined as follows: 1. Pain (*p* = 6.46 × 10^–9^), 2. Speaking (*p* = 1.86 × 10^–6^, 4. Swallowing, (*p* = 0.00811). 7. Eating (soft food) (*p* = 0.00167), and 8. Eating (seated) (*p* = 0.00475). Function was found to improve significantly after the use of the medication compared with that before use.

**Fig. 7:**
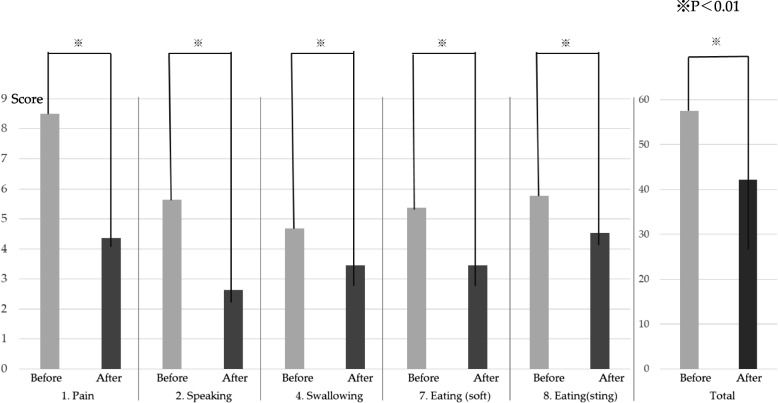
Comparative study of oral mucositis (OM) severity before and after the use of Episil®. The evaluation was completed using the Department of Oral Surgery, Hiroshima Red Cross & Atomic-bomb Survivors Hospital's original evaluation chart. Pain relief, deglutition function, feeding function, and overall function were significantly improved

No significant differences were observed in the function of eating hard foods; however, the functions of eating (soft food) and eating (stinging) showed significant improvement (Fig. 7). The overall score also showed a significant difference after the use of Episil® (*p* = 8.04 × 10^–7^) (Fig. 7).

### Study 2–2

Subsequently, Sections A and B of the department's original evaluation chart were examined. None of the items were found to have worsened after the use of Episil® compared with that before the use of Episil®. Although there was some variation among the items, (1) pain and (2) speaking showed a significant improvement in a large number of patients (Table 3). The *p*-values for (1) pain and (2) speaking were 5.483627 × 10^–6^ and 0.0262, respectively. In contrast, the *p*-values for the other items were > 0.86.

**Table 3:**
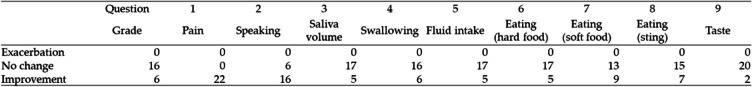
Study of the degree of improvement in each function with Episil®

In all the categories, the patients' performance did not decline after the drug was administered compared to their performance before taking the medication

In all the categories, the patients' performance did not decline after the drug was administered compared with their performance before taking the medication.

## Discussion

Building upon the efficacy of Episil® in treating OM among patients with hematologic malignancies. This study introduced a modification to the application method using "clean or gloved fingers”. Despite the package insert recommending that " spreading of Episil® on the affected area using the tongue," many patients were found to have difficulty in following this method. The method of application was modified in this study, as 26 of the 37 patients in Study 1–3 developed tongue mucositis, which made the application of Episil® with the tongue painful and difficult.

OM was observed at multiple sites in 23 (62.2%) of the 37 patients in Study 1–3. The presence of OM-related inflammation at multiple sites made it difficult to apply Episil® with the tongue, particularly in areas that were inaccessible to the tongue, such as the floor of the mouth and soft palate. Consequently, the use of different tools, such as cotton swabs, spoons, tongue depressors, and dental mirrors, was explored. However, the use of clean fingers was favored owing to the ease and cost-effectiveness of this method. As the use of Episil® was limited to hospitalized patients in this study, healthcare providers may have administered the drug when necessary, potentially influencing the study’s outcome. Patients were strictly prohibited from rinsing their mouths or eating or drinking for approximately 10 min after the application of Episil®, as the formation of the protective film occurred during this interval.

Cheng et al. [21] reported that the formation of the protective film after the application of Episil® occurred in < 1 min in 10% of patients, 1–5 min in 80% of patients, and > 5 min in 10% of patients. Ueno et al. [20] also noted that Episil® formed a protective film within 3–5 min of application in 80% of patients with head and neck cancer. Furthermore, they reported a decline in the mean numerical rating scale score starting at 5 min after Episil® application, with its duration of action lasting up to 120 min.

Hadjieva et al. [22] investigated the analgesic effect of Episil® in patients undergoing radiation therapy for head and neck cancer with OM. They reported that pain scores related to mucositis decreased rapidly 5 min after application, persisting for 8 h. The adhesive film was gradually removed via abrasion over time, indicating that its effects were not completely reduced after a single meal. These findings suggested that the effects of Episil® lasted long enough to support oral intake. Therefore, patients were encouraged to apply Episil® before meals and again after oral care. These instructions likely contributed to its effectiveness.

Study 1–2 and Study 1–3 revealed that 21% of patients experienced a decrease in the OM grade after the use of Episil®. Notably, none of the patients experienced exacerbations, indicating that Episil® was effective based on the subjective and objective evaluations of the patients. Additionally, the group showing improvement in grade exhibited a significantly higher mean number of sites where OM developed compared to the group with no change in grade. This result suggests that the formulation may be more effective in treating OM when multiple sites are affected.

Chemotherapy-induced mucositis occurs throughout the gastrointestinal tract. However, its incidence is particularly high in the oral cavity owing to the rapid turnover of basal cells in the oral mucosal epithelium, the presence of diverse bacterial flora, and their susceptibility to external stimuli, such as teeth, dentures, and food [23]. Moreover, OM serves as a gateway for pathogens to enter the body [24].

Nonetheless, only two studies have reported the use of Episil® for the treatment of OM in patients with hematologic malignancies [25, 26]. In the present study, comparisons of the OM grades in Study 1–2 and Study 1–3 suggest that Episil® is more useful for severe OM and OM affecting multiple sites. Wei et al. [27]. Reported a significantly lower incidence of severe OM (grades 3–4) after radiation therapy for head and neck cancer in the Episil® group compared to the control group. Therefore, Episil® may also be effective in reducing the severity of OM. Dentists may need to intervene early in patients with hematologic malignancies who are expected to develop severe neutropenia and consider oral care and the use of Episil®.

Study 2–1 assessed the patients’ evaluations of oral function and pain relief. The results revealed significant improvements in speech, swallowing, eating, and overall function scores after the use of Episil®. These findings suggest that the patient’s quality of life improved owing to pain relief as well as improved abilities in talking, eating, and swallowing. Previous studies that examined the use of Episil® and OM have focused on pain, feeding function, and nutritional status, and no literature regarding speech function is available, even in systemic reviews of OM [8–10].

Study 2–2 revealed that Episil® provided significantly higher improvements in pain relief and speech function than in other parameters. Interestingly, the present study revealed a novel observation indicating that the use of Episil® resulted in a greater degree of improvement in speech function compared to eating function. The improvement in speech function may have facilitated communication between patients and medical staff, contributing to the high patient satisfaction with Episil®. Moreover, the ability to talk also enhanced patients’ quality of life and treatment outcomes. Patients positive feedback on the use of Episil® was based on its efficacy in eliminating pain, enabling communication with healthcare providers and family members, and improving speech function.

Wei et al. [27]. reported a lower rate of weight loss in the Episil® group than in the control group at weeks 4 and 7 following radiotherapy for head and neck cancer. In addition, patients in the Episil® group had better body mass index and albumin levels and were less likely to be malnourished than those in the control group. The findings of the present study are consistent with these results, suggesting that Episil® improves nutritional status by relieving pain and enhancing feeding function.

According to the MASCC/ISOO guidelines [13], the number of studies focusing on treatment methods for OM has increased since 2014. However, these studies primarily focus on coping strategies, and notably, the use of Episil® is not mentioned in the revised 2019–2020 guidelines. Although the revised guidelines include a recommended protocol for oral photodynamic therapy (low-power laser therapy) for the prevention of OM, no devices have been approved in Japan for the prevention of OM [14].

Colella et al. [28] reported that OM is the most common and debilitating complication associated with the treatment of malignancies. Despite the significant clinical and economic consequences of this condition, there is little to offer patients with OM, and the medications used in its management are generally only palliative. Lalla et al. [29] also observed a scarcity of evidence supporting many approaches used for preventing, mitigating, and treating OM. They emphasized the need for additional long-term studies to develop accurate guidelines for the treatment and prevention of chemoradiotherapy-induced OM, given the scarcity of comprehensive data.

Palifermin, a recombinant human keratinocyte growth factor-1 (KGF-1), is the drug approved by the Food and Drug Administration (FDA) for the prevention of OM in patients receiving high-dose chemotherapy and systemic radiation therapy before HSCT [30]. The incidence of high-grade OM was found to be lower in patients receiving palifermin [31]. However, Pulito et al. [32] concluded that palifermin is not suitable for the treatment of OM owing to its high cost and concerns that the drug may sustain cancer cell growth.

Lalla RV et al. [29] focused on non-drug physical therapy for the treatment and prevention of OM and reported that low-intensity laser therapy and cryotherapy prevent the onset and reduce the duration of OM. Brown and Gupta [33] also reported that prophylactic dental interventions, such as restoration or extraction of carious teeth before commencing treatment, can reduce the risk of mucositis by approximately 25% and are particularly beneficial for patients at high risk of developing OM.

Lalla et al. [28] concluded that robust new methodological approaches, particularly in the human genome project, stem cell research, and complex data analysis, are needed. They also emphasized the necessity of forming new research teams with complementary expertise capable of leveraging the opportunities arising from these major research efforts and fostering interactions between these disciplines.

The prevention and treatment of OM are still in the developmental stage, and achieving a fundamental cure is a prolonged process. Palliative treatment will remain the mainstay for the foreseeable future. Despite Episil® appearing effective, several systematic reviews of OM have not yet considered it, emphasizing the need for further investigation.

The findings of this study suggest that Episil® is useful in treating OM in patients with hematologic malignancies. Only 37 patients were included in this study; therefore, we intend to conduct a multicenter study in the future to investigate and validate these results over time.

## Conclusions

Episil® was found to be effective in treating OM in patients with hematologic malignancies, particularly in those experiencing severe OM across multiple sites. In addition, Episil® proved effective in relieving pain and improving functions, such as speech and eating. These benefits could contribute to the overall treatment of the primary disease and improve the patients’ quality of life.

HSCT is the definitive treatment modality for hematologic malignancies, such as refractory leukemia. However, the unavoidable incidence of severe mucositis or mucositis involving the entire oral cavity due to pre-transplant chemotherapy poses a challenge. In this context, Episil® emerges as a potentially useful treatment option for OM in patients with hematologic malignancies, especially for those prone to severe neutropenia, where the use of topical steroid ointments is not feasible.

### Supplementary Information

**Supplementary Material 1.**

## Data Availability

The dataset supporting the conclusion of this article is available upon reasonable request from the corresponding author.

## Abbreviations

CTCAE: Common Terminology Criteria for Adverse Events
EMA: European Medicines Agency
FDA: Food and Drug Administration
GDO: Glycerol dioleate
OM: Oral mucositis
ROS: Reactive oxygen species
SPC: Soy phosphatidylcholine
