# Supplementary material for: Efficacy of Episil® in patients with hematologic malignancies: a comparative study
Source: BMC Oral Health. 2024 May 3;24:522. doi: 10.1186/s12903-024-04233-6 (PMC11067080; doi:10.1186/s12903-024-04233-6)
Supplement: Supplementary file 1 — Supplementary Material 1. [file 12903_2024_4233_MOESM1_ESM.docx]

**Efficacy of Episil® in patients with hematologic malignancies: a comparative study**

**Taeko Fukutani ^1*^, Yukio Yoshioka ^1^, Shinpei Imori ^2^, Hirokazu Yanagihara ^2^, Kensaku Sumi ^1^, Yoshinari Myoken ^3^ ,Yoshinori Fujita ^3^, and Souichi Yanamoto** ^1,^

^1^ Department of Oral Oncology, Graduate School of Biomedical and Health Sciences, Hiroshima University; [fukutani@hiroshima-u.ac.jp](mailto:fukutani@hiroshima-u.ac.jp), [yyosioka@hiroshima-u.ac.jp](mailto:yyosioka@hiroshima-u.ac.jp), [ksumi@horishima-u.ac.jp](mailto:ksumi@horishima-u.ac.jp), syana@hiroshima-u.ac.jp; Phone number: +81 (0)82 257-5667; Address; 1-2-3 Kasumi, Minami Ward, Hiroshima, 734-8553, JAPAN

^2^ Graduate School of Advanced Science and Engineering, Hiroshima University; [imori@hiroshima-u.ac.jp](mailto:imori@hiroshima-u.ac.jp), [yanagi-hiro@hiroshima-u.ac.jp](mailto:yanagi-hiro@hiroshima-u.ac.jp); Phone number: +81 (0)82 424-7357; Address; 1-3-2 Kagamiyama, Higashi-Hiroshima City, Hiroshima, 739-8511, , JAPAN 1-9-6

^3^ Department of Oral Surgery, Hiroshima Red Cross & Atomic-bomb Survivors Hospital; [myoken@do5.enjoy.ne.jp](mailto:myoken@do5.enjoy.ne.jp), yssnhfujita＠energy.ocn.ne.jp; Phone number: +81 (0)82 241-3111; Address; Sendamachi, Naka-ku,Hiroshima, 730-8619, JAPAN

***** Correspondence: fukutani@hiroshima-u.ac.jp; Tel.: +81-(0)-82-257-5667

**Supplementary Files**

**Table S1**. List of cases in Study 1
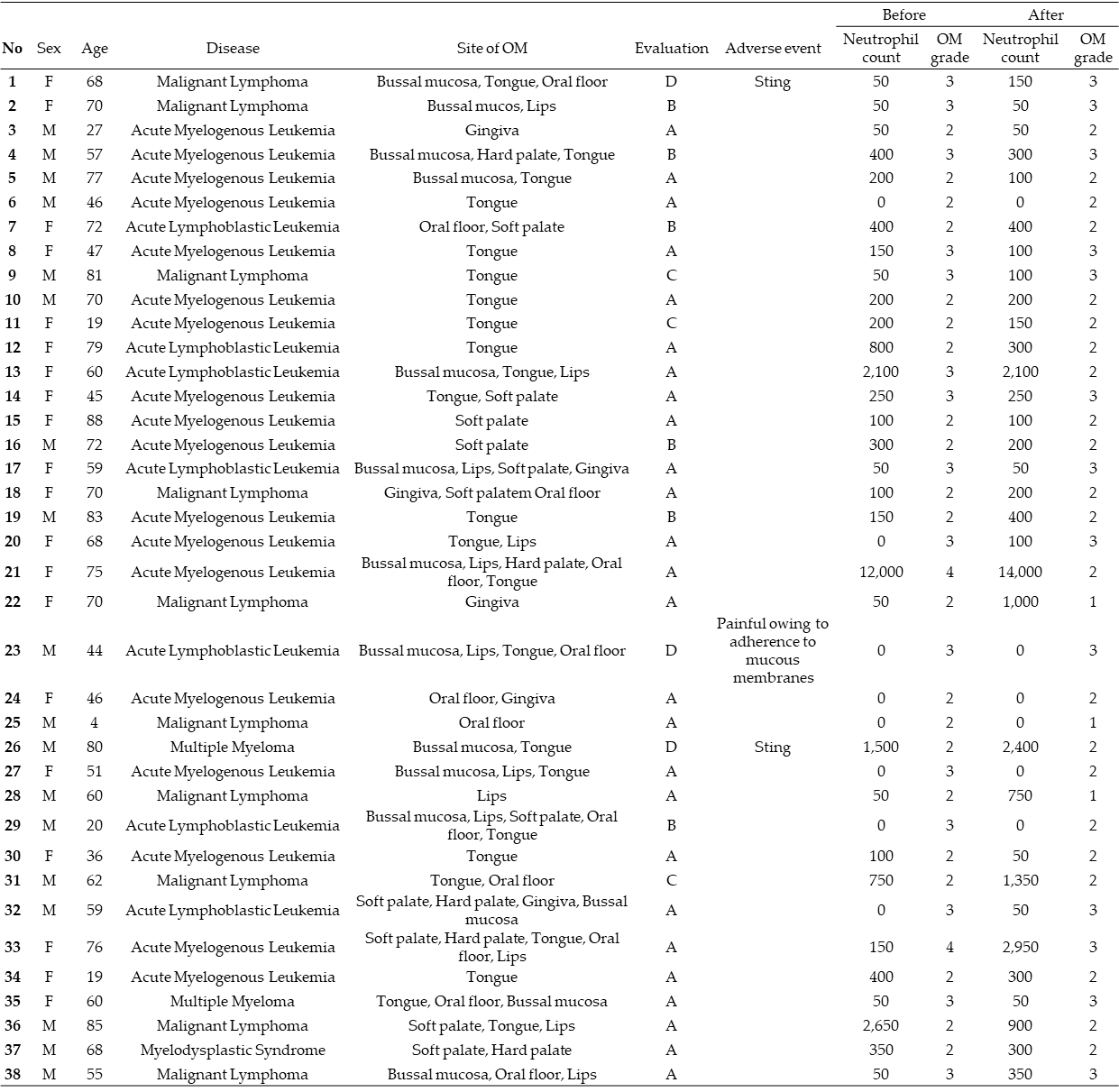


**Table S2.** List of cases in Study 2


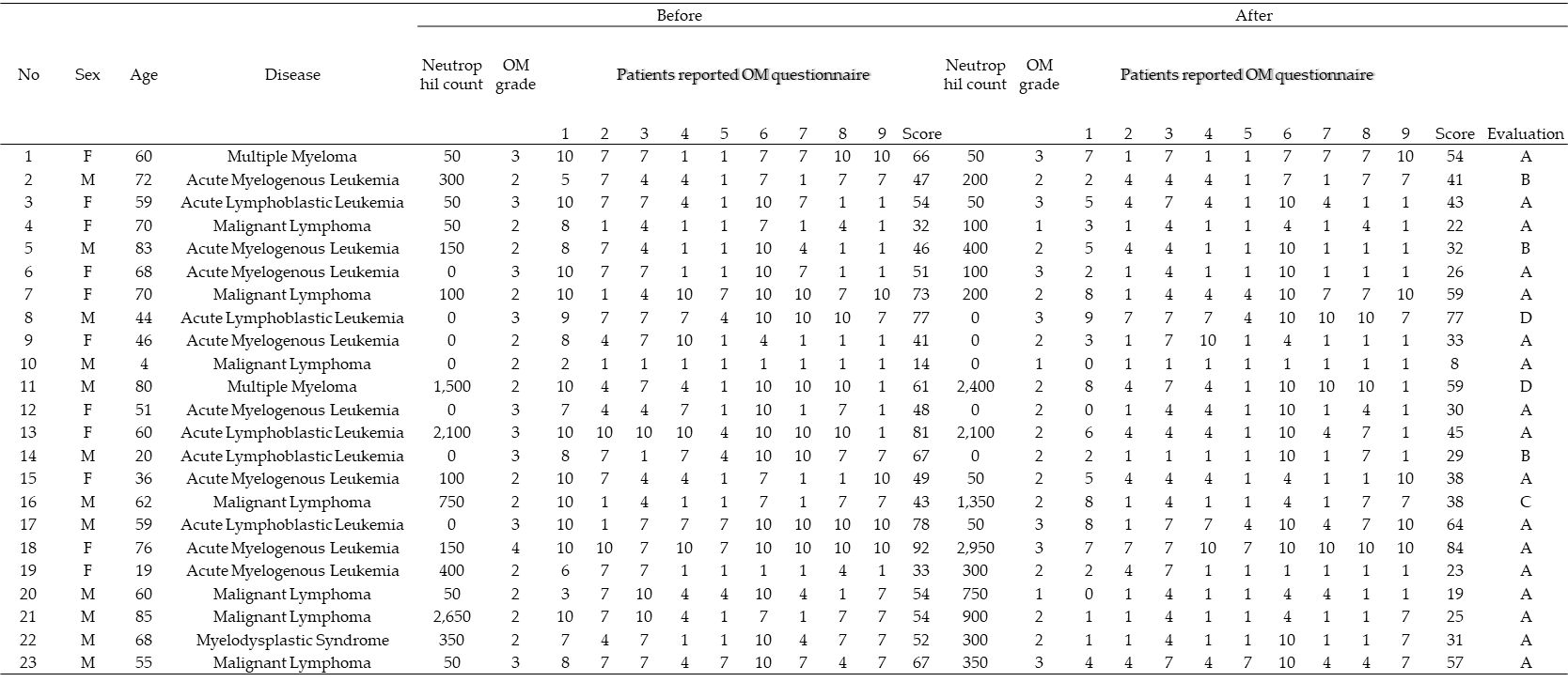


**Figure S1.** Lesion site of oral mucositis (OM). The tongue was the most common site affected by OM.
